# Supplementary material for: Discovery and application of insertion-deletion (INDEL) polymorphisms for QTL mapping of early life-history traits in Atlantic salmon
Source: BMC Genomics. 2010 Mar 8;11:156. doi: 10.1186/1471-2164-11-156 (PMC2838853; doi:10.1186/1471-2164-11-156)
Supplement: Additional file 2 — Information on developed 76 locus single-run INDEL panel in Atlantic salmon. Information on fluorescence labeling, primer concentrations, PCR pooling and links to alignments, INDEL motifs and GENESCAN (Burge and Karlin 1997) predictions of genes/exons are available in html format. [file 1471-2164-11-156-S2.ZIP › Additionalfile2/snpsummary17571.html]

```
Cluster 8478 Contig 1

prev  Summary    Contig List  next
```

Size of Consensus sequence = 776

Number of sequences = 6

Minimum redundancy = 2

Key

A gi|85046450|gb|DW574628.1|DW574628 EST\_ssal\_rgb2\_39047 rgb2 Salmo salar cDNA clone ssal\_rgb2\_563\_181\_fwd 3', mRNA sequence  
B gi|24352668|gb|CA046498.1|CA046498 ssalbrh015069 head Salmo salar cDNA, mRNA sequence  
C gi|117841345|gb|EG914041.1|EG914041 EST\_ssal\_evf\_54364 ssalevf mixed\_tissue Salmo salar cDNA Salmo salar cDNA clone ssal\_evf\_572\_326\_fwd 3', mRNA sequence  
D gi|89852204|gb|DY708327.1|DY708327 EST\_ssal\_rgb2\_64066 ssalrgb2 mixed\_tissue Salmo salar cDNA Salmo salar cDNA clone ssal\_rgb2\_603\_165\_fwd 3', mRNA sequence  
E gi|117841356|gb|EG914052.1|EG914052 EST\_ssal\_evf\_54365 ssalevf mixed\_tissue Salmo salar cDNA Salmo salar cDNA clone ssal\_evf\_572\_326\_rev 5', mRNA sequence  
F gi|24382558|gb|CA052315.1|CA052315 ssalrga507165 mixed\_tissue Salmo salar cDNA, mRNA sequence

9 SNPs detected

A B C D E F  cosegregation weighted

461 T - - T - T   9/9 100.00
462 T - - T - T   9/9 100.00
463 G - - G - G   9/9 100.00
464 T - - T - T   9/9 100.00
465 A - - A - A   9/9 100.00
466 A - - A - A   9/9 100.00
467 A - - A - A   9/9 100.00
468 A - - A - A   9/9 100.00
568 C G G C G C   9/9 100.00
